# Chest CT features of the novel coronavirus disease (COVID-19)

**DOI:** 10.3906/sag-2004-331

**Published:** 2020-06-23

**Authors:** Furkan UFUK, Recep SAVAŞ

**Affiliations:** 1 Department of Radiology, School of Medicine, Pamukkale University, Denizli Turkey; 2 Department of Radiology, School of Medicine, Ege University, İzmir Turkey

**Keywords:** COVID-19, pneumonia, chest computed tomography, radiation, diagnosis

## Abstract

A new type of coronavirus (2019-nCoV) is rapidly spreading worldwide and causes pneumonia, respiratory distress, thromboembolic events, and death. Chest computed tomography (CT) plays an essential role in the diagnosis of viral pneumonia, monitoring disease progression, determination of disease severity, and evaluating therapeutic efficacy. Chest CT can show important clues of 2019-nCoV disease (also known as COVID-19) in patients with an appropriate clinic. Prompt diagnosis of COVID-19 is essential to prevent disease transmission and provides close clinical observation of patients with clinically severe disease. Therefore, radiologists and clinicians should be familiar with the CT imaging findings of COVID-19 pneumonia. Herein, we aimed to review the imaging findings of COVID-19 pneumonia and examine the critical points to be considered for imaging in cases with COVID-19 suspicion.

## 1. Introduction

Since December 2019, a novel bat-origin coronavirus (2019-nCoV), also known as severe acute respiratory syndrome coronavirus 2 (SARS-CoV-2), capable of infecting humans has been identified, and the disease caused by this virus was officially named as COVID-19 by the World Health Organization [1]. Until April 27 2020, there were 2,804,796 confirmed cases with COVID-19 and 193,710 COVID-19 related deaths [2]. COVID-19 is a rapidly spreading viral disease and can cause serious health problems, especially pneumonia, necrotizing encephalopathy, systemic and pulmonary thromboembolism, acute respiratory distress syndrome (ARDS), respiratory failure, systemic inflammatory response, and sepsis [1,3–5]. Therefore, professional consensus, guidelines, and criteria have been published continuously to facilitate the diagnosis, management, follow-up, and isolation of patients [6–8].

Although the reverse transcription-polymerase chain reaction (RT-PCR) test is the gold standard for the diagnosis of COVID-19, chest x-ray and computed tomography (CT) have an essential role in the diagnosis, follow-up, and staging of COVID-19 pneumonia [3,5,9]. Chest CT may reveal parenchymal involvement in COVID-19 patients, including the areas of ground-glass opacities (GGO), interlobular septal thickening, prominent intralobular lines, and consolidation areas [9–14]. Moreover, especially in the early phases of the COVID-19 infection or in the presence of disease with a low viral load, the RT-PCR test may be negative, but CT may reveal significant findings [14,15]. However, in patients with COVID-19, especially in the early stage of the disease, chest CT can be found entirely normal, and normal CT does not rule out the disease. Moreover, chest radiography can be used in COVID-19 patients, but its diagnostic value is very limited. Therefore, it is recommended to use chest radiography in the assessment of lung involvement in paediatric and pregnant patients or in the follow-up of hospitalized patients, especially in case of severe or critical illness [16].

With growing global concerns about COVID-19 pandemic, radiologists and clinicians should be familiar with the imaging findings of COVID-19 pneumonia. Herein, we aimed to review the imaging findings of COVID-19 pneumonia and examine the critical points to be considered for imaging in cases with COVID-19 suspicion.

## 2. Usual imaging findings

The reported prevalence of the common CT findings is shown in Table 1.

**Table 1:** The reported prevalence of the usual computed tomography findings.

Publication	Number of patients	Study Origin	GGO	GGO with consolidation	Consolidation	Crazy paving pattern	Airbronchogram	Airway changes	PVE	Reticular and/or linear pattern
Xie et al. [9]	5	China	5 (100%)	2 (40%)	-	-	-	-	-	-
Fang et al. [10]	51	China	36 (72%)	-	-	-	-	-	-	-
Chung et al. [11]	21	China	12 (57%)	6 (29%)	0	4 (19%)	-	-	-	-
Bernheim et al. [13]	121	U.S.A.	41 (34%)	50 (41%)	2 (2%)	6 (5%)	-	16 (13%)	-	8 (7%)
Wu et al. [14]	80	China	73 (91%)	-	50 (63%)	23 (29%)	-	9 (11%)	-	16 (20%)
Song et al. [18]	51	China	39 (77%)	30 (59%)	28 (55%)	38 (75%)	41 (80%)	-	-	11 (22%)
Pan et al. [19]	63	China	54 (85.7%)	-	12 (19.0%)	-	-	-	-	11 (17.5%)
Ng et al. [20]	21	China	18 (86%)	-	13 (62%)	-	-	-	-	-
Pan et al. [21]	21	China	15 (71%)	-	19 (91%)	17 (81%)	-	-	-	-
Han et al. [22]	108	China	65 (60%)	44 (41%)	6 (6%)	43 (40%)	52 (48%)	-	86 (80%)	-
Xu et al. [23]	90	China	65 (72%)	-	12 (13%)	11 (12%)	7 (8%)	-	-	55 (61%)
Zhao et al. [24]	101	China	87 (86.1%)	65 (64.4%)	44 (43.6%)	-	-	53 (52.5%)	72 (71.3%)	49 (48.5%)
Zhou et al. [25]	62	China	25 (40.3%)	-	21 (33.9%)	39 (62.9%)	45 (72.6%)	20 (32.2%)	28 (45.2%)	35 (56.5%)
Xu et al. [26]	41	China	30 (73%)	25 (61%)	15 (37%)	33 (80%)	22 (54%)	-	-	-
Li et al. [27]	51	China	46 (90.2%)	28 (54.9)	3 (5.9)	36 (70.6)	35 (68.6)	-	42 (82.4)	10 (19.6)
Yang et al. [28]	149	China	-	-	-	-	81 (54.4%)	26 (17.4%)	-	79 (53%)
Ai et al. [29]	888	China	409 (46%)	-	447 (50%)	-	-	-	-	8 (1%)
Li et al. [30]	83	China	81 (97.6%)	-	53 (63.9%)	30 (36.1%)	-	19 (22.9%)	-	4 (4.8%)/ 54 (65.1%
Xiong et al. [31]	42	China	42 (100%)	-	23 (55%)	-	14 (33%)	-	-	15 (36%)
Bai et al. [32]	219	China	200 (91%)	141 (64%)	150 (69%)	11 (5%)	30 (14%)	19 (9%)	129 (59%)	123 (56%)/ 111 (51%)
Cheng et al. [33]	11	China	11 (100.0)	7 (63.6)	6 (54.5)	-	8 (72.7)	3 (27.3)	-	9 (81.8)
Shi et al. [34]	81	China	53 (65%)	-	14 (17%)	8 (10%)	38 (47%)	9 (11%)	-	3 (4%)
Wang et al. [35]	93	China	69 (74.2%)	-	56 (60.2%)	34 (36.6%)	-	44 (47.3%)	83 (89.2%)	15 (16.1)
Fan et al. [36]	150	China	124 (83%)	-	-	53 (35%)	54 (36%)	12 (8%)	-	-
Colombi et al. [37]	236	Italy	82 (35%)	119 (50%)	6 (3%)	-	-	-	-	-
Zhang et al. [38]	120	China	107 (89%)	-	62 (52%)	30 (25%)	24 (20%)	14 (12%)	-	22 (18%)
Zhu et al. [39]	72	China	36 (50%)	59 (82%)	16 (22%)	-	48 (67%)	-	33 (46%)	44 (61%)
Wang et al. [40]	110	China	30 (27.3%)	50 (45.4%)	30 (27.3%)	-	-	-	-	-
Li et al. [41]	56	China	45 (80.4%)	43 (76.8%	12 (21.4%)	25 (44.6%)	41(73.2%)	-	-	30 (53.6%)
Guan et al. [42]	47	China	47 (100%)		30 (64%)	42 (89%)	36 (77%)	-	-	-
Liu et al. [43]	67	China	50 (75%)	-	8 (12%)	28 (42%)	-	19 (28%)	-	-

GGO; ground-glass opacity, PVE; pulmonary vascular enlargement.

### 2.1. Ground glass opacity

**Figure 1:**
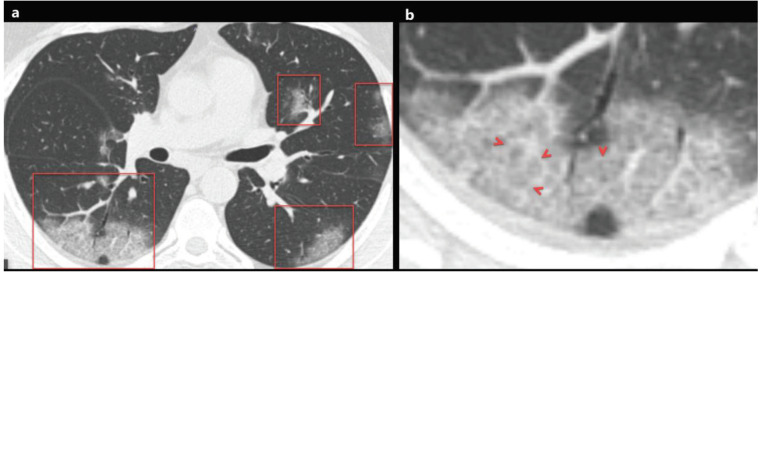
a) A 48-year-old man with COVID-19 presented with cough, fever, myalgia, and malaise for 3 days. Axial chest CT image shows multifocal, peripheral-peribronchovascular ground-glass opacities (red frames). b) The magnified image of the right lower lobe shows ground-glass opacity superimposed with interlobular septal thickening and prominent intralobular lines (red arrowheads), which indicates a crazy-paving pattern.

Ground glass opacity (GGO) is an increase in lung density without obscuring vascular margins in the lung parenchyma because of partial filling of air spaces, interstitial lung disease, increased capillary blood volume, partial alveolar collapse, or a combination of these, the common factor being the partial displacement of air [17]. Ground glass opacities with a peripheral and multi-lobar distribution is a common finding in COVID-19 patients, and GGO is usually more prominent in lower-middle lobes and posterior lung areas (Figures 1a and 1b). The reported prevalence of GGO varies between 46% and 100%, and GGO has usually seen in the early phases of the disease and/or mild pulmonary infection [9–11,13,14,18–43]. As the disease stage and severity increase, usually consolidation and/or interstitial thickening (crazy paving pattern) begins to appear within the GGO areas. Besides, while GGO or consolidation are healing, it can be seen as a GGO containing fibrotic band or atelectasis in the lung [31].

### 2.2. Consolidation

**Figure 2:**
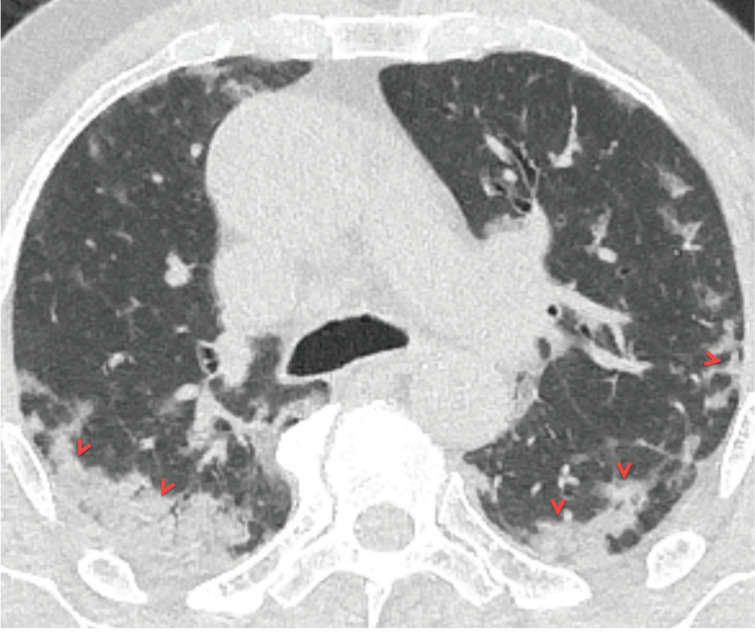
A 66-year-old symptomatic man with COVID-19 pneumonia. Axial CT image at the carina level shows peripheral ill-defined consolidation areas in the both middle-lower lung zones with subpleural involvement (red arrowheads). Note the “air bronchogram sign” within the consolidation areas.

Pulmonary consolidation is defined as an increase in lung density with obscuring vascular margins in the lung parenchyma due to the complete replacement of air spaces (alveolus and alveolar sacs) by fluid, pus, blood, or cells [17]. Peripheral, segmental, or sub-segmental consolidation areas with the multi-lobar distribution are usually seen in cases with COVID-19 pneumonia, especially in older patients and patients with severe or critical disease (Figure 2) [11,14–16,31,39]. Consolidation areas are usually seen in mixed patterns with GGO, and the new emergence of consolidation areas in the follow-up imaging of COVID-19 patients was considered as an indicator of progressive disease [11]. Besides, consolidative lesions have been reported in COVID-19 patients with a longer time interval between onset of symptoms and chest CT scan, or patients over 50 years of age [31,39]. The prevalence of thromboembolic events in patients with COVID-19 has been shown to increase compared to the healthy population. Therefore, when CT shows peripherally located, triangular-shaped consolidation areas, pulmonary thromboembolism, and infarction (Hampton hump) should be considered in the differential diagnosis [44,45].

### 2.3. Crazy paving pattern

The crazy paving pattern indicates a ground-glass opacity (GGO) with superimposed interlobular septal thickening and prominent intralobular lines [14,17]. Crazy paving pattern opacities are not uncommon in COVID-19 patients, and the reported prevalence of crazy paving pattern varies between 5% and 89% [9–11,13,14,18–43] (Figures 1a and 1b) (Table 1). Crazy paving pattern may indicate interstitial inflammation and alveolar damage in patients with COVID-19, which had been previously reported in patients with Middle East Respiratory Syndrome (MERS) and the Severe Acute Respiratory Syndrome (SARS) [46]. In the follow-up, the increase in the density of GGO and the emergence of opacities with the crazy paving pattern was demonstrated in the early stage of COVID-19 pneumonia. Moreover, the progression of the crazy paving pattern to consolidation and the emergence of pleural effusion have been reported in the late stage of COVID-19 pneumonia [8,19,31].

### 2.4. Air bronchogram and airway changes

**Figure 3:**
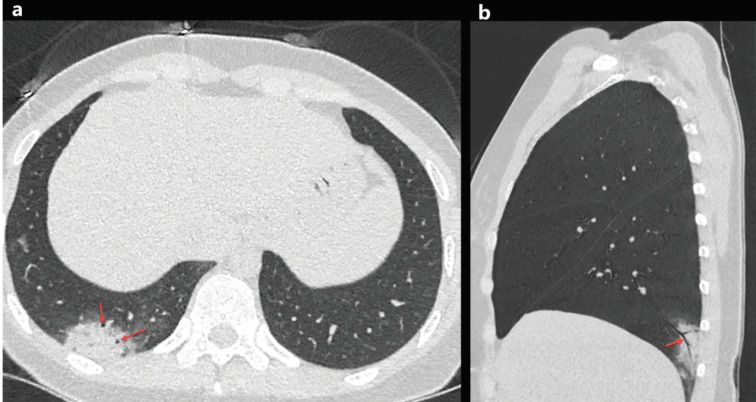
A33-year-old woman with COVID-19 presented with cough, fever, and pleuritic chest pain for 2 days. Axial chest CT image shows a focal consolidation with air bronchogram sign in the right lower lobe (a, red arrows). The sagittal reconstructed CT image of the right lower lobe shows air bronchogram sign inside the consolidation (b, red arrow).

Air bronchogram is the appearance of air-filled and low attenuated bronchi on a background of the high attenuated opaque lung, and the air bronchogram has almost always been caused by airspace pathology [17]. Air bronchogram sign has been frequently reported in cases with COVID-19 pneumonia (Figures 3a and 3b). Song et al. [18] have been reported that 80% (n = 41/51) of patients with COVID-19 pneumonia had air bronchograms. Besides, COVID-19 patients may have airway changes such as endobronchial mucus plugging, bronchiectasis, bronchioloectasis, and bronchial wall thickening [9–11,13,14,18–43] (Table 1). Moreover, the prevalence of bronchial wall thickening in COVID-19 patients with severe-critical clinical features has been shown to be significantly higher than in patients with mild-common clinical features [14,41].

### 2.5. Pulmonary vascular enlargement

**Figure 4:**
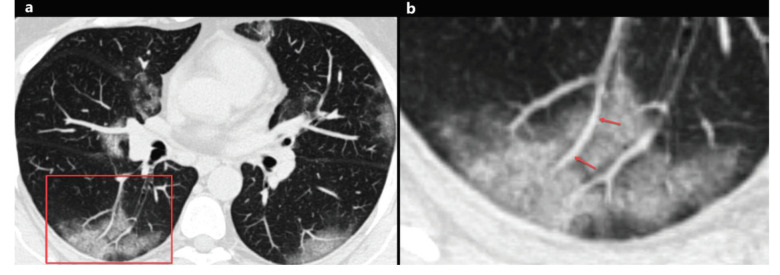
A 46-year-old woman with COVID-19. Axial chest CT image shows peripheral and peribronchovascular distributed focal ground-glass opacities in both lungs, and focal vascular enlargement is seen inside the ground-glass opacity in the right lower lobe (a, red frame). The magnified image of the red frame shows vascular enlargement inside the ground-glass opacity (b, arrows).

Pulmonary vascular enlargement (PVE), also known as pulmonary vascular widening, is defined as the widening of the subsegmental pulmonary vessel (pulmonary arteries and veins) with a diameter of more than 3 mm around and/or inside the opacity on CT (Figures 4a and 4b) [24,25,27,32,35,39]. Although the pathophysiology of PVE is not fully understood, PVE plays a potential diagnostic role for COVID-19. Bai and colleagues [32] had compared the CT findings in patients with COVID-19 pneumonia and non-COVID-19 pneumonia, and they found that the PVE was significantly associated with COVID-19 (P < 0.001, 59% vs. 22%). Moreover, PVE was reported in 45.2% to 89.2% of COVID-19 patients [24,25,27,32,35,39] (Table 1). PVE can be attributed to the vascular wall inflammatory infiltration [24,32,35].

### 2.6. Reticular pattern and linear opacification

**Figure 5:**
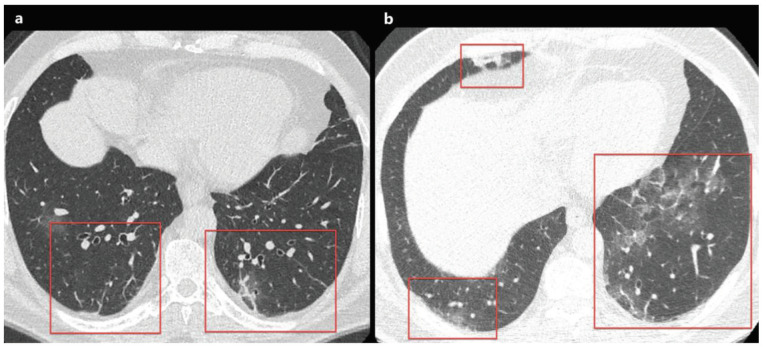
a) A 66-year-old symptomatic man with COVID-19. Axial CT image shows subpleural fibrous stripes with ground-glass opacities in both lower lobes (red frames). b) A 53-year-old man with COVID-19. Axial CT image shows subpleural curvilinear lines, interlobular septal thickening, and fibrous stripes with ground-glass opacities in both lower lobes (red frames).

The reticular pattern is the pathological process of the pulmonary interstitium and characterized by interlobular septal thickening and prominent intralobular lines [17]. The reticular pattern is usually seen in COVID-19 patients with pneumonia and reticulation may increase in patients with longer disease course [8,19,31]. The reported prevalence of reticular pattern and linear opacification is very variable in the literature and has been reported between 1% and 81% [13,14,18,19,23–41]. Moreover, subpleural curvilinear lines and fibrous stripes have been reported in COVID-19 patients (Figures 5a and 5b) [14,18–43], and both may represent the replacement of cellular components by fibrosis [14,19]. It has been shown that cured patients with COVID-19 pneumonia may have persistent reticular opacities on CT [31].

### 2.7. Pulmonary embolism

**Figure 6:**
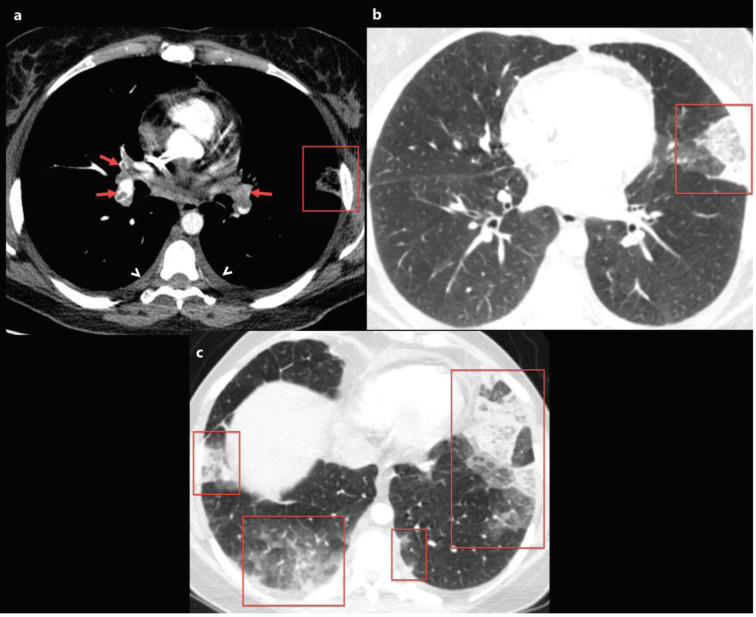
A 40-year-old female patient was presented with complaints of fever that had been ongoing for 5 days, and newly emerged severe dyspnoea. The patient’s D-dimer level was found to be very high and contrast-enhanced pulmonary CT angiography was obtained. a) Axial CT image at the level of the origin of the middle lobe bronchus with mediastinum window settings shows bilateral diffuse pulmonary embolism (red arrows), bilateral mild pleural effusion, and subpleural triangular-shaped opacity with a reverse halo sign in the left lung compatible with pulmonary infarction (red frame). b, c) Axial CT images of the same patient with lung window settings show multiple subpleural consolidation and ground-glass opacity areas (red frames). The nasopharyngeal swab test of the patient was positive for 2019-nCoV.

Life-threatening thromboembolic events are increasingly identified in COVID-19 patients [44,45,47]. Recently, Grillet and colleagues [44] reported the prevalence of pulmonary embolism (PE) as 23% (n = 23/100) in COVID-19 patients with severe clinical features, and they reported that COVID-19 patients with PE, had a higher requirement for mechanical ventilation than those without PE. Moreover, Leonard-Lorant et al. [47] reported that 22 of 106 patients (30%) with COVID-19 had acute pulmonary thromboembolism. Elevated D-dimer levels have been frequently reported in COVID-19 cases, especially in COVID-19 patients with PE [5,45,47]. Current guidelines recommend performing unenhanced chest CT for COVID-19 suspected patients with risk factors and/or severe-critical clinical findings. However, thromboembolic events associated with COVID-19 infection are not uncommon. Besides, especially those treated in the intensive care unit and patients in mechanical ventilation therapy are in the high-risk group for deep venous thrombosis and PE [44,45]. Therefore, pulmonary CT angiography is essential for evaluating lung parenchyma and vascular complications for COVID-19 cases with severe and/or critical clinical features and high D-dimer levels (Figures 6a, 6b, and 6c). Recently, the British Thoracic Imaging Association has suggested that if pulmonary CT angiography (CTA) is required in a potential COVID-19 patient, prior to CTA, an unenhanced chest CT should be performed because mosaic attenuation in CTA can be confused with GGO [48].

## 3. Unusual imaging findings

The reported prevalence of the unusual CT findings is shown in Table 2.

**Table:** The reported prevalence of the unusual computed tomography findings.

Publication	Number of patients	StudyOrigin	Pleural thickening and/or effusion	Air bubble sign	Nodules	Reversedhalo sign	Spiderweb sign	LAP	Pericardial effusion
Chung et al. [11]	21	China	0 (0%)	-	0 (0%)	-	-	0 (0%)	-
Bernheim et al. [13]	121	U.S.A.	1 (0.8%)	-	0 (0%)	2 (1.7%)	-	0 (0%)	-
Wu et al. [14]	80	China	5 (6%)	-	-	-	20 (25%)	3 (4%)	4 (5%)
Song et al. [18]	51	China	4 (8%)	-	-	-	-	3 (6%)	3 (6%)
Pan et al. [19]	63	China	-	-	8 (12.7%)	-	-	-	-
Ng et al. [20]	21	China	0 (0%)	-	1 (4.8%)	2 (9.6%)	-	0 (0%)	0 (0%)
Pan et al. [21]	21	China	-	-	-	-	-	0 (0%)	-
Han et al. [22]	108	China	0 (0%)	-	-	-	-	0 (0%)	0 (0%)
Xu et al. [23]	90	China	4 (4%)	-	-	-	-	1 (1%)	1 (1%)
Zhao et al. [24]	101	China	14 (13.9%)	-	23 (22.8%)	-	-	1 (1%)	-
Zhou et al. [25]	62	China	6 (9.7%)	34 (54.8%)	-	-	-	0 (0%)	-
Xu et al. [26]	41	China	2 (7.1%)	-	-	-	-	1(3.6%)	-
Li et al. [27]	51	China	1 (2.0 %)	-	11 (21.6%)	2 (3.9 %)	-	0 (0%)	-
Yang et al. [28]	149	China	10 (6.7%)	12 (8.1%)	3 (2%)	-	-	7 (4.7%)	-
Ai et al. [29]	888	China	-	-	24 (3%)	-	-	-	-
Li et al. [30]	83	China	7 (8.4%)	-	6 (7.2%)	-	21 (25.3%)	7 (8.4%)	4 (4.8%)
Xiong et al. [31]	42	China	5 (12%)	-	-	-	-	12 (29%)	0 (0%)
Bai et al. [32]	219	China	32 (15%)	-	70 (32%)	11 (5%)	-	6 (3%)	-
Cheng et al. [33]	11	China	0 (0%)	1 (9.1)	3 (27.3)	-	-	0 (0%)	-
Shi et al. [34]	81	China	4 (5%)	8 (10%)	5 (6%)	-	-	5 (6%)	-
Wang et al. [35]	93	China	8 (8.6%)	12 (12.9%)	17 (18.3%)	14 (15.1%)	-	6 (6.5%)	-
Fan et al. [36]	150	China	6 (4%)	-	18 (12%)	-	-	2 (1.3%)	-
Colombi et al. [37]	236	Italy	47 (20%)	-	-	-	-	57 (24%)	-
Zhang et al. [38]	120	China	9 (8%)	-	65 (54%)	-	-	5 (4%)	-
Zhu et al. [39]	72	China	3 (6.8)	36 (50%)	-	-	-	-	-
Wang et al. [40]	110	China	1 (0.9%)	-	-	-	-	-	-
Li et al. [41]	56	China	5 (8.9%)	-	0 (0%)	-	-	0 (0%)	-
Guan et al. [42]	47	China	0 (0%)	-	1 (2.1%)	-	-	0 (0%)	-
Liu et al. [43]	67	China	3 (4.5%)	-	-	-	-	-	-

LAP; lymphadenopathy.

### 3.1. Pleural thickening and effusion

**Figure 7:**
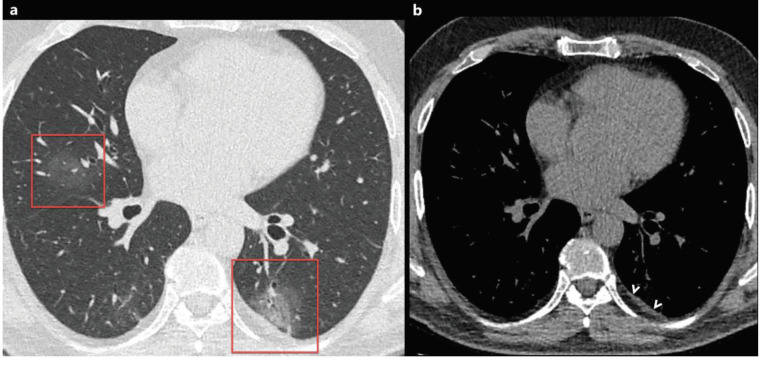
A 56-year-old man with COVID-19. Axial chest CT image with lung window settings shows bilateral focal ground-glass opacities in both lungs (a, red frame). Chest CT image with mediastinum window settings shows focal pleural thickening in the left lower lobe, near the focal ground-glass opacity (b, arrowheads).

Pleural pathologies such as pleural effusion and focal pleural thickening have been rarely reported in cases with COVID-19, and pleural pathologies are usually seen in the later stages of the disease [21,31,39]. The presence of pleural effusion is thought to be a sign of poor prognosis in COVID-19 pneumonia, and the prevalence of pleural effusion in COVID-19 patients has been reported to range from 0% to 20% [11,13,14,18–43] (Table 2). Besides, the pleural effusion and thickening might be associated with dense pleural inflammation in patients with COVID-19 (Figures 6a, 6b, 6c, 7a, 7b) [24]. 

### 3.2. Air bubble sign

**Figure 8:**
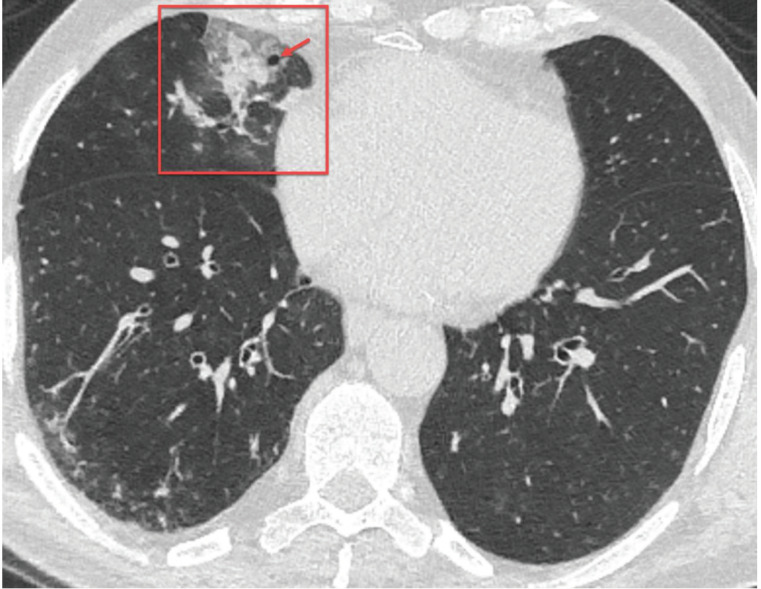
A 75-year-old symptomatic man with COVID-19. Axial chest CT image shows mixed opacity (ground-glass opacity with consolidation) area in the right middle lobe (red frame), and that includes cystic air space (air bubble, red arrow).

The air bubble sign has been defined as a small air-containing space that may be a pathological enlargement of bronchioles inside the opacity [25,28]. Air bubble sign is named in the literature under different names (cystic air space, round cystic changes, air-containing space, sieve-hole sign, and vacuolar sign). Although its pathophysiology is unclear, it may be related to the pathological expansion of alveolar sacs or bronchioles or the absorption process of consolidation, and the reported prevalence of air bubble sign in patients with COVID-19 pneumonia ranges from 8.1% to 54.8% (Figure 8) [25,28,33, 34, 35,39] (Table 2). 

### 3.3. Nodules

**Figure 9:**
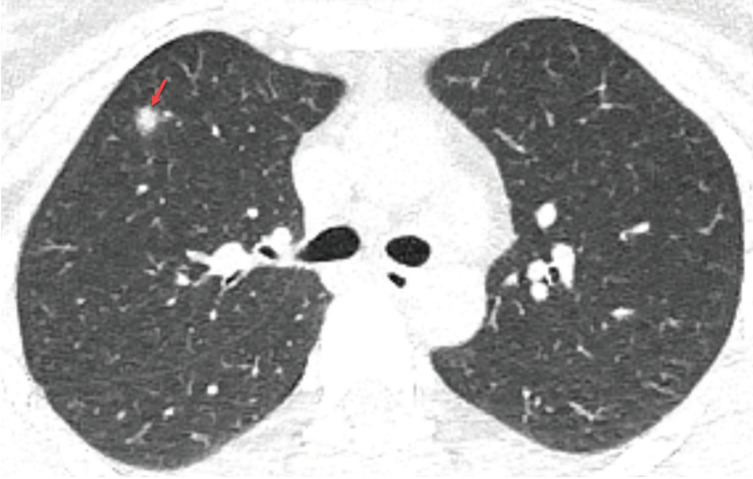
A 36-year-old woman presented with complaints of cough and fever for 2 days. Axial chest CT image shows a nodule with halo sign in the right upper lobe (red arrow). The nasopharyngeal swab test of the patient was positive for 2019-nCoV, and the nodule disappeared at follow-up.

A nodule refers to opacity in the lung parenchyma, less than or equal to 3 cm in diameter [17]. Shi and colleagues [34] reported that lung nodules were detected in 6% of patients with COVID-19 pneumonia. Similarly, Li and colleagues [30] reported that pulmonary nodules with or without visible halo sign were detected in 7.2% of patients with COVID-19 pneumonia (Figure 9). The halo sign indicates GGO surrounding a pulmonary nodule or mass and usually seen in patients with invasive fungal infections, hypervascular pulmonary metastases, and organizing pneumonia [17]. Although it is reported in the literature that tree-in-bud pattern nodules can be seen in patients with COVID-19 [24,28, 33,35], superimposed bacterial infections or aspiration should be considered first when these nodules are detected.

### 3.4. Reversed halo sign 

**Figure 10:**
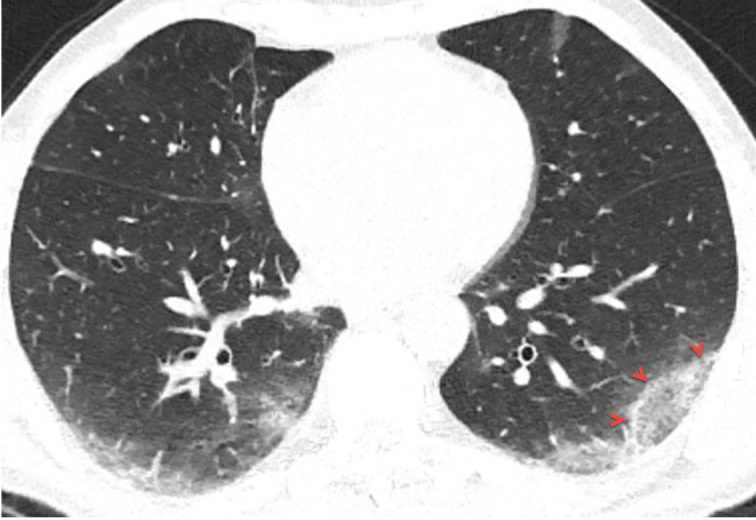
A 58-year-old man with COVID-19. Axial chest CT image with lung window settings shows bilateral multifocal ground-glass opacities in both lungs. The ground-glass opacity in the left lower lobe surrounded by denser ring-like (crescentic shape) consolidation, which is compatible with a reversed halo sign (arrowheads).

The reversed halo sign (RHS) indicates a central GGO surrounded by denser ring-like (crescentic shape) consolidation and also known as the Atoll sign [17]. Although RHS is usually seen in patients with cryptogenic organized pneumonia (COP), it has been reported in several cases with COVID-19 [13,20,27,32,35]. While Bernheim et al. [13] reported that the reversed halo sign was detected in 2 of 121 patients (1.7%) with COVID-19 pneumonia, Wang et al. [35] reported the prevalence of reversed halo sign as 15.1% (14 of 93 patients) (Table 2) (Figure 10).

### 3.5. Spider web sign

The “spider web sign” was defined by Wu and colleagues [14], and they described it as an angular or triangular-shaped peripheral GGO with interstitial thickening like a spider web in a corner, but its pathophysiology has not been clearly revealed. They reported that the reversed halo sign was detected in 20 of 80 patients (25%) with COVID-19 pneumonia [14]. Moreover, Li et al. [30] detected the spider web sign in 21 of 83 (25.3%) patients with COVID-19 pneumonia. Apart from these 2 studies, there are no other studies in the literature describing or investigating the “spider web sign”. Therefore, this finding needs to be investigated in patients with non-COVID-19 pneumonia and COVID-19 pneumonia.

### 3.6. Lymphadenopathy

Mediastinal lymphadenopathy has been infrequently reported in COVID-19 patients, and lymphadenopathy has been shown as a risk factor for severe and progressive COVID-19 pneumonia [14,31,37]. In the literature, the frequency of mediastinal lymphadenopathy has been reported between 0% and 29% in patients with COVID-19 [11,13,14,18–38]. Lymphadenopathy is a rare finding in viral pneumonia, and the detection of pleural effusion, lung nodules with a tree-in-bud pattern, and ill-defined micronodules may suggest bacterial superinfection [31–34].

### 3.7. Pericardial effusion

Pericardial effusion has been rarely reported in COVID-19 patients, which may indicate the occurrence of myocardial and/or pericardial inflammation [14,18,23,30]. Li et al. [30] have reported that COVID-19 patients with severe and critical diseases showed a higher frequency of pericardial effusion than noncritical patients. Recently, Xu et al. [23] found the pericardial effusion in 1 of 90 (1.1%) patients with COVID-19 pneumonia. However, elderly patients, and especially those with additional heart failure, may have pericardial effusion independent of COVID-19.

## 4. Structured CT reporting for assessment of COVID-19

Although chest CT findings in COVID-19 patients can partially overlap with other diseases, especially other types of viral infections, COVID-19 may have characteristic CT features that are less common in other diseases [32]. Various recommendations have been published to standardize the structured CT reporting for the suspected COVID-19. The first of these is the Radiological Society of North America (RSNA) expert consensus statement, which suggests a standardized reporting and a classification of imaging features for COVID-19 pneumonia (i.e. negative for typical appearance, vague appearance, atypical appearance, and pneumonia) [49]. Salehi and colleagues [50] have defined a new classification by analysing 37 published studies that examined the diagnostic chest CT findings of COVID-19 patients. There are 5 categories in this classification: COVID-RADS 0; normal CT findings, COVID-RADS 1; atypical CT findings (inconsistent with COVID-19), COVID-RADS 2A; fairly typical findings, COVID-RADS 2B; combination of atypical findings with typical/fairly typical findings, and COVID-RADS 3; typical CT findings [50] (Table 3). However, the groups in this classification are based on the results obtained from a systematic review, and clinical success and applicability of this classification are unknown. Recently, the Dutch Radiological Society defined a new classification (CO-RADS) for pulmonary involvement in cases presenting with moderate to severe symptoms of COVID-19 [51]. In this standardized assessment (CO-RADS), a substantial agreement was found among 8 observers (Fleiss’ kappa of 0.47, 95% CI 0.45–0.49) and the discriminatory power of the CO-RADS for diagnosing COVID-19 was high (with an area under the curve value of 0.91, 95% CI 0.85–0.97). Therefore, the authors stated that CO-RADS is very suitable for use in clinical practice [51] (Table 4).

**Table 3:** The proposed coronavirus disease 2019 (COVID-19) imaging reporting and data system (COVID-RADS) [50].

CT findings	Description	COVID-RADS grade	Level of suspicion
Normal chest CT		0	Low
Atypical findings	- Pleural effusion	1	Low
(Inconsistent with COVID-19)	- Cavity		
	- Pulmonary nodule(s)		
	- Nodular pattern		
	- Lymphadenopathy		
	- Peribronchovascular distribution		
	- Halo sign		
	- Tree-in-bud sign		
	- Bronchiectasis		
	- Airway secretions		
	- Pulmonary emphysema		
	- Pulmonary fibrosis		
	- Isolated pleural thickening		
	- Pneumothorax		
	- Pericardial effusion		
Fairly typical findings	- Single GGO (early)	2A	Moderate
	- Consolidation without GGO (late/complicated)		
	- Focal pleural thickening		
	- Vascular enlargement		
	- Air bronchogram		
	- Bronchial wall thickening		
	- White lung stage (late/complicated)		
	- Parenchymal fibrotic bands (late/remission)		
Combination of atypical findingswith typical/fairly typical findings		2B	Moderate
Typical findings	- Multifocal GGO	3	High
	- GGO with superimposed consolidation		
	- Consolidation predominant pattern		
	(late/complicated)		
	- Linear opacities (late/complicated)		
	- Crazy paving pattern (late/complicated)		
	- Melted sugar sign (late/remission)		

GGO, ground glass opacity.

**Table 4:** The proposed CO-RADS categories and the corresponding level of suspicion for pulmonary involvement in COVID-19 [51].

	Level of suspicion for pulmonary involvement of COVID-19	Summary
CO-RADS 0	Not interpretable	Scan technically insufficient for assigning a score
CO-RADS 1	Very low	Normal or noninfectious
CO-RADS 2	Low	Typical for other infection but not COVID-19
CO-RADS 3	Equivocal/unsure	Features compatible with COVID-19, but with also other diseases
CO-RADS 4	High	Suspicious for COVID-19
CO-RADS 5	Very high	Typical for COVID-19
CO-RADS 6	Proven	RT-PCR positive for SARS-CoV-2

## 5. The severity of pulmonary involvement on CT

The visual (semiquantitative) evaluation of disease severity on chest CT can reflect the clinical classification (mild, common, severe, or critical disease) and prognosis of patients with COVID-19 pneumonia [11,30,41]. Therefore, the staging of lung involvement has been defined in COVID-19 patients. In these staging methods, the percentage of each lung lobe involvement is assessed, and the portion of the total lung involvement is calculated semiquantitatively (visually). For example, Chung et al. [11] and Li et al. [41] used the same scoring as follows: none (0%); a score of 0, minimal (1%–25%); a score of 1, mild (26%–50%); a score of 2, moderate (51%–75%); a score of 3, and severe (76%– 100%); a score of 4. An overall lung involvement score was reached by summing the 5 lobe scores (0–20). Besides, quantitative CT assessment methods and artificial intelligence applications can be successfully applied in the diagnosis of COVID-19 and in the evaluation of disease severity [37,52,53].

## 6. Radiation concern

Exposure to medical radiation is a concern in patients with suspected or diagnosed COVID-19 pneumonia, especially in infants, children, young adults, and pregnant patients. Therefore, it is necessary to prevent or reduce radiation exposure [6,7,20,54]. First, it should be distinguished appropriately in which patients need imaging and in which patient imaging is not needed. In cases with COVID-19 suspicion, imaging methods should be avoided if clinical follow-up and management will not change. In a multinational recommendation that is newly defined and widely used by Fleischner Society, imaging is not recommended for cases with suspected of COVID-19 without risk factors (such as diabetes, older age, chronic obstructive pulmonary disease) and mild clinical findings. Moreover, imaging methods are indicated in a COVID-19 patient whose respiratory condition has worsened [6]. Second, chest CT should be obtained as low as possible radiation dose in cases with CT indication. Dangis et al. [54] showed that chest CT with low-dose settings would provide the diagnosis of COVID-19 pneumonia with similar accuracy as standard-dose chest CT.

## 7. Contamination of radiology units

Radiography machines and CT scanners are frequently used in patients with suspected COVID-19 during the pandemic. Therefore, vital precautions should be provided for COVID-19 in radiology units [6,55]. First, ensuring the health of radiology workers is essential for the best care of patients. For this reason, staff working in radiology units should use necessary personal protective equipment according to their contact with the patient. Besides, all patients with a suspicion for COVID-19 who presented to the radiology unit should use a standard surgical mask. Second, contamination of radiography machines and CT scanners are a major concern. Therefore, it is necessary to use a dedicated radiography machine and CT device in patients with suspicion for COVID-19 to reduce the risk of disease transmission. Also, these dedicated devices should never be used in patients without suspicion for COVID-19. Third, to reduce the risk of contamination, the CT scanners and radiography devices and rooms must be deeply cleaned and disinfected according to standard protocols after each exam. If patient masking is available, active (mechanically) air exchange procedures are not required [6,55]. After each imaging procedure, the room downtime for the decontamination of room and devices is approximately 30 min.

## 8. Conclusion

In conclusion, early diagnosis of COVID-19 is essential to prevent transmission and provide close clinical observation of the patients. While the gold standard for the diagnosis of COVID-19 is RT-PCR, CT should be obtained in a limited population (in COVID-19 patients who have worsening respiratory symptoms, in patients with moderate-severe clinical features of COVID-19, in medical triage of COVID-19 suspected cases with moderate-severe clinical features, and a high pretest probability of COVID-19 in the lack of personal protective equipment or availability of RT-PCR test) due to radiation concern. Although bilateral and peripheral GGO without or with superimposed interstitial thickening (crazy-paving pattern), and/or consolidation with the multi-lobar distribution is the common CT features in COVID-19, chest CT features can vary among patients. Moreover, chest CT is not just a diagnostic or screening method in cases with suspected COVID-19. In patients with COVID-19, chest CT is especially crucial in determining the severity of pneumonia, which has a prognostic impact [11,37,41]. 

## Acknowledgements

Authors declare that there is no conflict of interest. No informed consent was received.
